# Elucidating the combined effect of intermittent hypoxia training and acetazolamide on hypoxia induced hematological and physiological changes

**DOI:** 10.1016/j.crphys.2022.07.004

**Published:** 2022-07-18

**Authors:** Megha A. Nimje, Himadri Patir, Rajeshkumar Tirpude, Bhuvnesh Kumar

**Affiliations:** a Defence Institute of Physiology and Allied Sciences (DIPAS), (DRDO), Lucknow Road, Timarpur, Delhi, 110054, India; b Sharda University, Greater Noida, Uttar Pradesh, 201310, India

**Keywords:** Intermittent hypoxia training, Acetazolamide, Oxygen desaturation, High altitude acclimatization

## Abstract

As the number of people travelling to altitude increases, the risk of life threatening medical emergencies also increases. It is important that we have effective strategies to minimize the risk of altitude illness. In this study, an attempt was made to investigate the combined effect of non-pharmacological (Intermittent hypoxia training; IHT) and pharmacological (acetazolamide; ACZ) intervention as a prophylactic strategy in order to minimize the risk of high altitude hypoxic related problems using rats as an animal model. Male Sprague Dawley rats were subjected to IHT for 4 h consecutively for 5 days at 12% FiO_2_ under normobaric conditions with and without oral ACZ administration at 25 mg/kg body weight. Validation of the intervention was performed by exposing the rats to extreme hypoxia (EH) at 8% FiO_2_ to further assess the effect of IHT and ACZ on hypoxic acclimatization. The principal findings of this study is that the combined effect of IHT and ACZ improves the arterial oxygenation by alterations in hemodynamics and in blood gasometry, thereby resulting into an increase in the oxygen carrying capacity of the blood with increase in SpO_2_ (peripheral oxygen saturation). The present study showed that the combined effect of IHT with ACZ could be refined as a prophylactic measure for better outcomes during altitude ascent and rapid altitude acclimatization rather than IHT or ACZ alone.

## Highlights

• Combination of IHT with ACZ attenuates the inhibitory effect of respiratory alkalosis.
• It also minimizes pathological changes in hematology and blood gas.
• The strategy improved the oxygen carrying capacity of the blood by increasing SpO_2_.
• Thus, it leads to ventilatory Acclimatization.
• The combined treatment could be refined as a prophylactic measure for altitude maladies.

## 1 Introduction

Gradual and effective adaptation to high altitude (HA) environment is essential for sojourners to prevent high altitude associated illnesses like acute mountain sickness (AMS) and related pathologies. Initial and most apparent response to HA is hyperventilation (Moore et al., 1986; Muza et al., 1995) followed by hypocapnia and increase in alveolar as well as arterial oxygenation. Various interventions have been used by mountaineers which one way or other, initiate the process of altitude acclimatization or inhibit harmful effects of HA environment.

The carbonic anhydrase inhibitor, acetazolamide is one of the most frequently used drug, as a preventive measure for AMS as demonstrated initially by Forwand et al. (1968), followed by a large study on Everest trekkers (Hackett et al., 1976). Acetazolamide has its primary action on kidney leading to metabolic acidosis and bicarbonate dieresis (Leaf and Goldfarb, 2007). Metabolic acidosis, in turn, attenuates the inhibitory effects of hypoxia induced respiratory alkalosis (Leaf and Goldfarb, 2007) and thereby rapidly imitates the biological process of ventilatory acclimatization (West, 2004). However, dose related adverse effects have been observed in case of acetazolamide treatment. Meta analysis of acetazolamide drug dose to prevent AMS is found to be 250, 500, and 750 mg/day (Ritchie et al., 2012). Systematic review on acetazolamide intake showed that dose related adverse effects such as risk of taste disturbance and polyuria increased with 500 and 750 mg/day dosage (Kayser et al., 2012; Ritchie et al., 2012). Although 250 mg daily dose or 125 mg twice daily of acetazolamide is the lowest effective dose to reduce the incidence and severity of AMS effectively (Basnyat et al., 2006; Ritchie et al., 2012), it may also lead to side effects such as paraesthesia of extremities and other side effects including drowsiness, nausea, myopia, and an increased risk of kidney stones (Low et al., 2012). Furthermore, acetazolamide is a sulfonamide drug, therefore, the general recommendation is that patients with known allergies to sulfa drugs should avoid acetazolamide (Schoene, 2008). People with history of severe penicillin allergy have occasionally had allergic reactions to acetazolamide (Schoene, 2008). However, Platt and Griggs (2012) reported the safe and successful use of acetazolamide for treatment of patients with episodic ataxia and periodic paralysis who had a history of severe allergic reactions to antibiotic sulfonamides. Despite the adverse effects of acetazolamide, it has been reported as altitude sickness medicine in several studies (Hudalla et al., 2019; Luks et al., 2019; Ritchie et al., 2012; Swenson, 2014).

Intermittent Hypoxia training (IHT), on the other hand, is considered as a non pharmacological intervention for high altitude related health problems in humans (Bhaumik et al., 2018; Muza, 2007). Intermittent hypoxia (IH) can be defined as periodic exposure to hypoxia lasting for minutes to days, interrupted by return to normoxia or less hypoxic conditions (Powell and Garcia, 2000). Intermittent hypoxia training is postulated to improve oxygenation at higher altitude by increasing hypoxic chemosensitivity and ventilation (Katayama et al., 2001). Intermittent hypoxia has shown many beneficial roles such as attenuation of central fatigue during exercise in severe hypoxia (Amann et al., 2013), increasing whole body exercise tolerance in severe hypoxia (Twomey et al., 2017) and recovering muscle damage due to eccentric exercise by modulating many mitochondrial biomarkers in skeletal muscles (Rizo-Roca et al., 2017). Intermittent hypoxia exposure programs are also applied in the field of sports medicine to improve aerobic capacity (Ricart et al., 2000). Improvement in exercise performances in athletes as well as defense against certain diseases has been extensively studied using IHT (Fulco et al., 2000; Marzatico et al., 1986). Intermittent hypoxia also induced anti-arrhythmic effect in acute myocardial ischemia (Meerson et al., 1989) and prevented experimental atherosclerosis in different animal models (Kitaev et al., 1999). Studies with severe hypoxic exposure of 2–8% FiO_2_ had detrimental cardiovascular, respiratory, cognitive and metabolic outcomes while low-dose hypoxic exposure of 9–16% FiO_2_ had positive therapeutic outcomes in humans treated for chronic clinical disorders (Navarrete-Opazo and Mitchell, 2014). Therefore, the question remains unclear that under which circumstances, advantages of intermittent hypoxia treatment outweigh the long term risks of extreme hypoxia exposures.

Acetazolamide is known to support acclimatization. Whereas, IHT has recently been discussed as a potential strategy for acclimatization before going to high altitude. Burtscher et al. (2022) proposed a short IHT schedule of as low as 7 h for pre-acclimatization to high altitude. The authors suggested that hypobaric hypoxia exposures were more efficient in pre-acclimatization to high altitude exposures, however owing to technical reasons and easy to use system, normobaric hypoxia exposure is more common. Nonetheless, as discussed by Hilty et al. (2020) several research questions still remain open due to the diverse research methodologies. The safety of the protocol and the optimal protocol remains uncertain.

Evidences from the previous study reports reveal that both acetazolamide and IHT was found to improve SpO_2_ levels individually (Basnyat and Murdoch, 2003; Nimje et al., 2020). However, the mode of action of IHT and acetazolamide was found to be contrasting. It was reported that IHT works by enhancing the sensitivity of peripheral chemoreceptors (Bao et al., 1997; Katayama et al., 2001) while, acetazolamide has been shown to have inhibitory effects on the peripheral chemoreceptors and as reported in several of the studies conducted on cats (Teppema et al., 1988, 2001, 2006).

In Brief, based on the discussion of literature from previous findings as well as the present study, a hypothesis is proposed that combined treatment of Intermittent hypoxia training (IHT) and Acetazolamide (ACZ) might serve as a efficient prophylactic strategy for high altitude acclimatization by inducing the following mechanism. Hyperventilation caused by hypoxia will reduce the alveolar pCO_2_ by increasing the rate of clearance of CO_2_ from the alveoli and thus decreasing the partial pressure of CO_2_ (hypocapnia). Increased clearance of CO_2_ from the pulmonary circulation will lead to hypoxia induced respiratory alkalosis, resulting in acid-base imbalance. Respiratory alkalosis and hypocapnia in turn will inhibit hyperventilation through a negative feedback on the respiratory centre. Combined administration of IHT and ACZ might attenuate the hypoxia induced respiratory alkalosis through bicarbonate (HCO3^-^) diuresis, reduced base excess (BE) thereby reducing pH. Parallel to this, pathological changes in hematological variables viz., WBC, RBC, Hb, HCT, reticulocyte, NRBC will be reduced due to effect of IHT. All these will further result in ventilatory acclimatization resulting in increased SpO_2_, increased respiratory rate and stabilized heart rate.

Therefore an attempt was made in the present study to investigate the combined effect of non-pharmacological (IHT) and pharmacological (Acetazolamide) approaches as a novel strategy for enhancing high altitude acclimatization.

## 2 Methods

### 2.1 Experimental model of the study

**Fig. 1:**
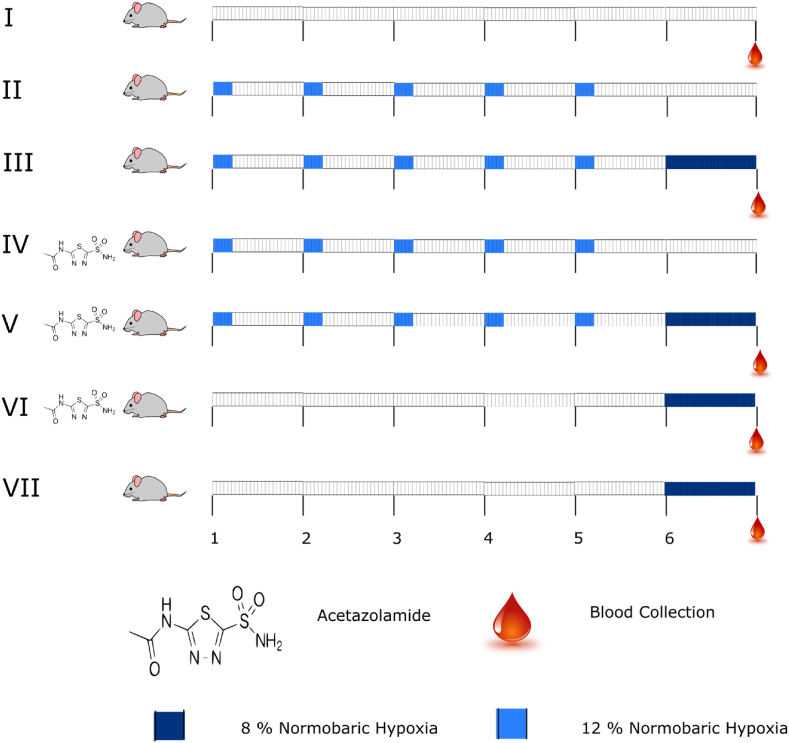
Schematic representation of blood collection schedule. A total of 35 animals were divided into seven groups (n = 5). Animals were grouped as following; Group-I: Normoxia (N); Group-II: Intermittent hypoxia training (12% FiO2 for 4 h consecutively for 5 days) (IHT); Group-III: Group B followed by Extreme hypoxia exposure at 8% FiO2 (IHT + EH); Group-IV: IHT + Acetazolamide administration (25 mg/kg of body wt) (IHT + ACZ); Group V: Group IV followed by EH (IHT + ACZ + EH); Group-VI: Acetazolamide administration followed by EH (ACZ + EH); Group-VII: Extreme hypoxia exposure at 8% FiO_2_ (EH).

The study was conducted using male Sprague Dawley rats as an animal model with an average body weight of 180 ± 10 g. To further rule out the prevalence of commonly found infestations, rats obtained from the experimental animal facility of the institute were further screened for respiratory infectious agents viz., Sendai virus (SMART-R11), Corona virus (SMART-R20) and Mycoplasma pulmonis (SMART-R27) using the commercially available kits (Biotech Trading Partners, USA). From the colony of screened animals a total of 35 apparently healthy animals were selected and divided into seven groups (n = 5). Animals were grouped as follows; Group-I: Normoxia (N); Group-II: Intermittent hypoxia training (12% FiO2 for 4 h consecutively for 5 days) (IHT); Group-III: Group II followed by Extreme hypoxia exposure at 8% FiO_2_ (IHT + EH); Group-IV: IHT + Acetazolamide administration @ 25mg/kg of body wt. (IHT + ACZ); Group V: Group IV followed by EH (IHT + ACZ + EH); Group-VI: Acetazolamide administration followed by EH (ACZ + EH); Group-VII: Extreme hypoxia exposure at 8% FiO2 (EH). Rats were given light anesthesia with Pentobarbital @ 30 mg/kg body wt. in order to collect blood to perform further experiments. The blood collection schedule is depicted schematically in the Fig. 1.

All animal procedures and experimental protocols were approved by the Institutional Animal Ethics Committee (IAEC) of the institute (IAEC Number: DIPAS/IAEC/2019/05) and followed the standards outlined in the Committee for the Purpose of Control and Supervision of Experiments on Animals (CPCSEA), Animal Welfare Board, Ministry of Agriculture, Government of India.

### 2.2 Acetazolamide drug dosage

The drug acetazolamide was purchased from Sigma-Aldrich (St. Louis, MO, USA). Previous studies in human subjects have shown that prophylactic administration of acetazolamide at a dose of 125 mg twice daily was the lowest effective dosage in inducing a significant decline in AMS symptoms (Lipman et al., 2019). Acetazolamide drug dosage for rats was extrapolated mathematically as described by Shin et al. (2010).

Briefly, human equivalent dose (HED) calculations were based on body surface area. Thus, Animal dose (mg/kg) = HED (mg/kg) X Conversion factor. Where, HED Acetazolamide = 250 mg twice daily, the conversion factor for rat as calculated by dividing km factor of Human (37) by km factor of rats (6) is 6.17. Assuming average adult of 70 kg, Acetazolamide dose per kg body weight is 3.571 mg (250mg/70 kg).

Hence, rat acetazolamide dose (mg/kg) = 3.571 mg/kg × 6.17 = 22.03 ∼25 mg/kg body wt.

Accordingly, daily dose of acetazolamide @ 25 mg/kg body wt. was prepared freshly by dissolving in 0.05% dimethyl sulfoxide (DMSO) and administered orally, 1 day before the start of IHT and 1 h before the IHT session each day. Drug dosage was maintained below the Oral LD50 of 4300 mg/kg (Acetazolamide sc-214461, SDS).

### 2.3 Intermittent hypoxia training (IHT)

In the present study, intermittent hypoxia exposures of the animals were performed in a normobaric hypoxia chamber (Hypoxicator-Jarvis 50 LN, Biostag Technologies, India) by reducing the percentage of the fraction of inspired oxygen (FiO_2_).

The rats were exposed to normobaric hypoxia at 12% FiO_2_ (equivalent to 14,000 ft) for 4 h consecutively for 5 days as schedule established in our previous study (Nimje et al., 2020).

#### 2.3.1 Validation studies

For validating the collective role of IHT with acetazolamide in altitude acclimatization, rats were further exposed to 8% FiO_2_ (equivalent to 25,000 ft) for 6 h under normobaric conditions. This study time point of 6 h duration for validating the IHT was followed based on the observations made in our previous study, where rats exposed to extreme hypoxia for 6 h duration resulted into an increase in edema and inflammation of the lung tissues of the rats (Nimje et al., 2020).

Normobaric chamber was maintained at an optimum temperature (25 ± 1 °C) and humidity (50–60%), throughout the experiment. Animals were provided with food and water *ad libitum*.

### 2.4 Monitoring of physiological parameters (SpO_2_, heart rate and respiratory rate)

Peripheral capillary oxygen saturation (SpO_2_), heart rate (HR) and respiratory rate (RR) of conscious unanesthetized rats were measured throughout the duration of hypoxia exposure in normobaric chamber through pulse oximeter neck collar clip sensors (MouseOx plus, STARR Life Sciences, Oakmont, PA, USA). Data were integrated in real-time through multichannel data recording unit (Powerlab, AdInstruments). Recorded data were stored and analyzed on LabChart software (AdInstruments). In order to obtain precise data, rats were habituated to neck collar clip by placing a demonstration clip on the back of the neck daily two weeks prior to actual experimentation.

### 2.5 Hematological studies

Blood was collected from retro-orbital plexus with the help of anti-coagulant coated capillaries following light anesthesia (pentobarbital; 30 mg/kg body wt.). Blood was collected in EDTA vacutainers (BD) and immediately analyzed through 5-part differential veterinary hematology analyzer (Spincell vet5 Compact, Spinreact, Spain). Whole blood routine CBC and retics test were performed.

### 2.6 Blood gas analysis

Blood was collected from the retro-orbital plexus of rat under light anesthesia through heparinised capillary tubes and was allowed to drop directly into the i-STAT CG8+ cartridge in order to avoid minimum contact of the blood sample with air, and was analyzed immediately through i-STAT analyzer (Abbott, East Windsor, N. J. USA). Following parameters viz., blood pH, blood gas composition (PCO_2_- partial pressure of Carbon Dioxide, PO_2_- partial pressure of oxygen in venous blood) and blood electrolyte (BE-Base Excess, HCO_3_^−^ –bicarbonate) were measured.

### 2.7 Statistical analysis

Results were expressed as mean ± SD. Data was assessed by one-way analysis of variance (ANOVA) applying the Bonferroni correction for multiple comparisons among groups. Differences were considered statistically significant at p < 0.05. All statistical tests were performed with Graphpad PRISM, Version 6.01.

## 3 Results

### 3.1 Changes in physiological variables during IHT

Changes in average physiological variables during the 4 h IHT session at 12% FiO_2_ are depicted in Table 1.

**Table 1:** Changes in physiological variables during Intermittent hypoxia training.Values are mean ± SD (n = 5). Table represents the average values of the changes in physiological variables during the 4 h IHT session at 12% FiO_2_. Ns, non-significant difference.

Day of IHT	SpO_2_ (%)		RR (brpm)		HR (bpm)	
	IHT	IHT + ACZ	IHT	IHT + ACZ	IHT	IHT + ACZ
1	65.17 ± 3.44	64.37 ± 3.81 ^ns^	120.97 ± 17.34	132.81 ± 14.54*	403.59 ± 39.97	442.15 ± 40.65 *
2	69.4 ± 3.54	74.02 ± 3.114 ^$^	129.12 ± 13.14	147.70 ± 16.89 ^$^	429.21 ± 14.23	443.36 ± 44.89 ^ns^
3	74.36 ± 5.16	77.39 ± 4.46 *	120.54 ± 12.71	130.56 ± 10.29 *	394.72 ± 41.458	420.02 ± 30.90 *
4	68.93 ± 3.96	72.65 ± 4.76 *	122.86 ± 14.52	124.46 ± 16.94 ^ns^	439.17 ± 11.57	429.37 ± 31.60 ^ns^
5	69.74 ± 5.13	72.56 ± 4.7 ^ns^	120.22 ± 6.59	127.05 ± 21.05 ^ns^	366.38 ± 40.61	412.84 ± 39.38 ^$^

*p < 0.05, #p < 0.01, $p < 0.0001. SpO2% (Peripheral Oxygen Saturation), RR (Respiratory Rate), HR (Heart Rate).

#### 3.1.1 Oxygen saturation (SpO_2_)

Vital physiological variables were monitored prior to experimentation (21% FiO_2_) to establish control values. At 21% FiO_2_, oxygen saturation was observed to be 95.57 ± 2.40%. Combined effect of IHT and ACZ maintained a higher level of SpO_2_ throughout the intermittent hypoxia training sessions (Table 1).

#### 3.1.2 Respiratory Rate (RR)

Baseline RR value at sea level was 94.63 ± 15.02brpm. Respiratory rate was found to be increased in all the groups during the IHT session. Respiratory rate was found to be significantly increased in the combined IHT and ACZ group on the 1st, 2nd and 3rd day of IHT as compared to the IHT alone group with a non-significant difference on the 4th and 5th day of IHT (Table 1).

#### 3.1.3 Heart Rate (HR)

Baseline HR value at sea level was 334.93 ± 41.11bpm. Heart rate was found to be increased in all the groups during the IHT session. It was found to be significantly increased (p < 0.0001) in the combined IHT and ACZ group as compared to the IHT alone group by the 5th day of IHT (Table 1).

### 3.2 Validation studies

After IHT sessions of 12% FiO_2_ for 4 h consecutively for 5 days, validation studies for the effect of IHT was performed on the 6th day by exposing the rats to extreme hypoxia at 8% FiO_2_ for 6 h duration. Changes in physiological, hematological and blood gas variables were monitored, after the stipulated time period of 6 h extreme hypoxia exposure.

#### 3.2.1 Physiological variables

##### 3.2.1.1 Oxygen saturation (SpO_2_)

**Fig. 2:**
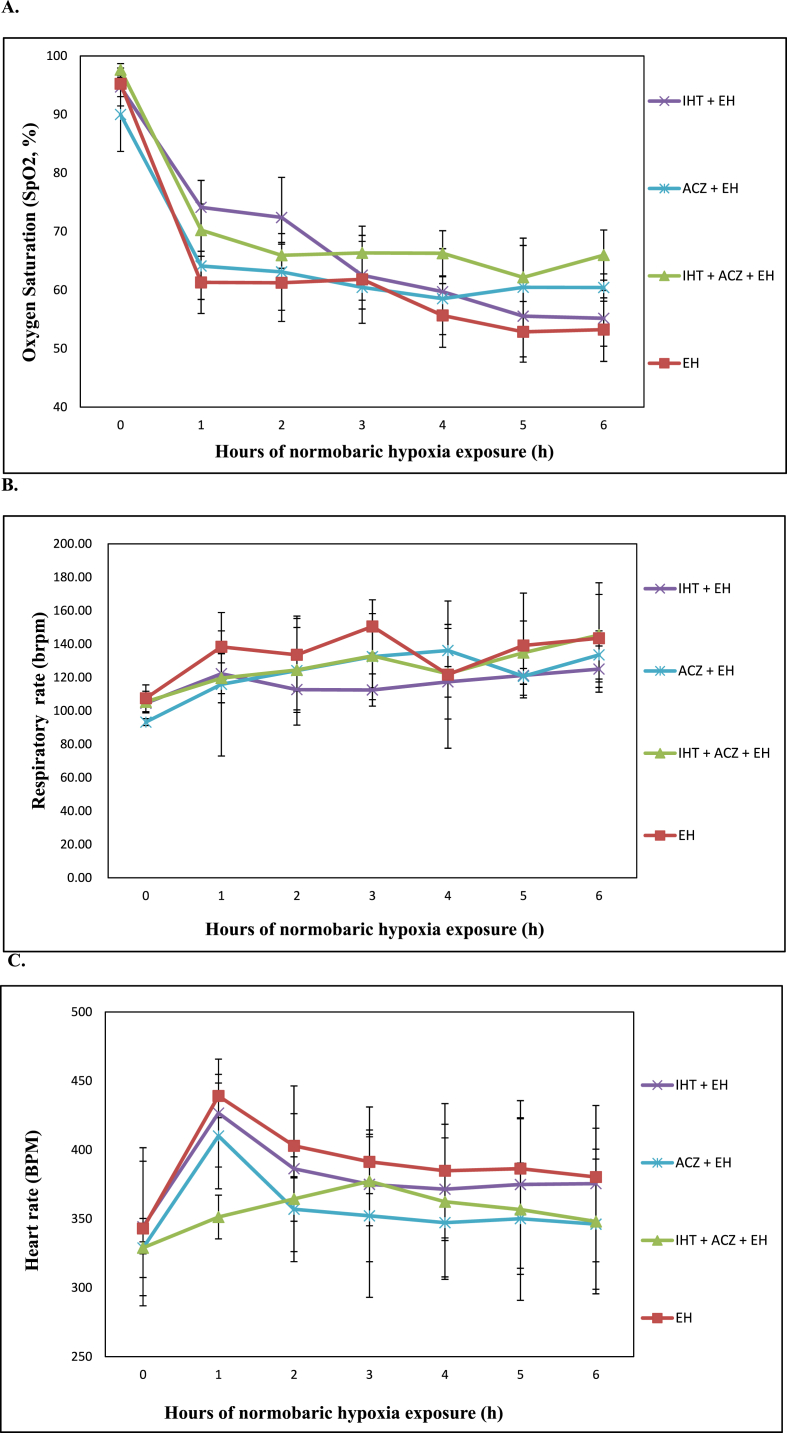
Validation study of intermittent hypoxia training at extreme hypoxia exposure at 8% FiO_2_ on day 6th of IHT. Values are mean ± SD (n = 5). a. Effect of Intermittent Hypoxia Training on peripheral capillary oxygen saturation (SpO2%) recorded during the 6 h exposure session. b. Effect of Intermittent Hypoxia Training on Heart Rate (bpm) recorded during the 6 h exposure session. c. Effect of Intermittent Hypoxia Training on Respiratory Rate (brpm) recorded during the 6 h exposure session.

Validation study was carried out at extreme hypoxia exposure of 8% FiO_2_ on day 6th after IHT exposures. Exposure to extreme hypoxia at 8% FiO_2_ resulted into a steep decline in the level of SpO_2_% in all the hypoxia exposed groups. However, with increasing time period it maintained a horizontal SpO_2_% in the last hours of exposure (Fig. 2 A). The combined effect of IHT and ACZ maintained a higher and constant SpO_2_% throughout the duration of extreme hypoxia exposure.

##### 3.2.1.2 Respiratory rate (RR)

Changes in RR during the extreme hypoxia exposure duration were found to be arrhythmic in all the groups (Fig. 2 B). Peak level of RR was observed in the extreme hypoxia exposed group reaching to a peak level at 3^rd^ h of hypoxia exposure (150.53brpm), followed by ACZ administered extreme hypoxia exposed group at 4^th^ h of hypoxia exposure (136.12brpm).

##### 3.2.1.3 Heart rate (HR)

Validation study at extreme hypoxia exposure at 8% FiO_2_ was observed with increasing HR during the initial FiO_2_ decrease (Fig. 2 C). Unlike other groups, combined IHT and ACZ group showed a slow and gradual rise in the HR reaching to a peak level at 3^rd^ h of the hypoxia exposure (377.23bpm).

### 3.3 Changes in hematological parameters

**Fig. 3:**
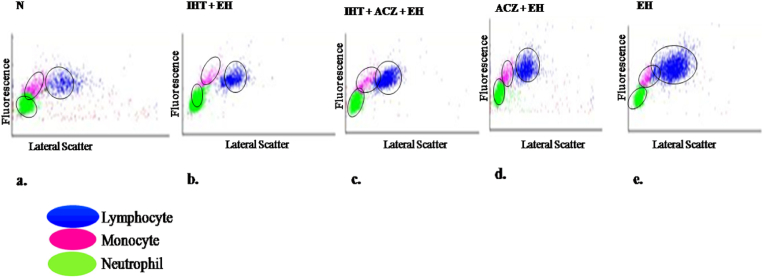
Differential leukocyte expression depicted in scattergram of the rats exposed to 8% FiO_2_ analyzed through fluorescence based automated cell counter. a) Normoxia, b) IHT + EH (Intermittent hypoxia training + Extreme hypoxia), c) IHT + ACZ + EH (Intermittent hypoxia training + Acetazolamide + Extreme hypoxia), d) ACZ + EH (Acetazolamide + Extreme hypoxia), e) EH (Extreme hypoxia).

**Fig. 4:**
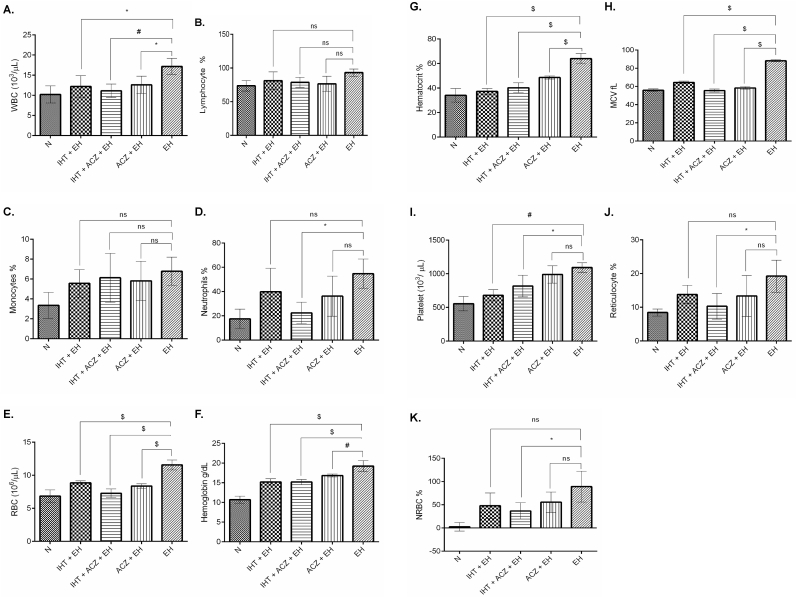
Graph representing differential expression of hematological values. Data was assessed by one-way analysis of variance (ANOVA) applying the Bonferroni correction for multiple comparisons among groups. Values are mean ± SD, n = 5. N: Normoxia, IHT + EH: Intermittent hypoxia training + Extreme hypoxia, IHT + ACZ + EH: Intermittent hypoxia training + Acetazolamide + Extreme hypoxia, ACZ + EH: Acetazolamide + Extreme hypoxia, EH: Extreme hypoxia. **A.** WBC; White Blood Cells (10^3^/μl), **B.** Lymphocytes (%), **C.** Monocytes (%), **D.** Neutrophils (%), **E.** RBC; Red Blood Cells (10^6^/μl), **F.** Hemoglobin (g/dL), **G.** Hematocrit (%), **H.** MCV; Mean Corpuscular Volume (fL), **I.** Platelet (10^3^/μl), **J.** Reticulocyte %, **K.** NRBC; Nucleated red blood cells %. *p < 0.05, #p < 0.001, $p < 0.0001.

Hematological changes after exposure to extreme hypoxia at 8% FiO_2_ were characterized by a significant increase in WBC, lymphocytes, monocytes, neutrophils, RBC, HCB, HCT, MCV, PLT, Retic, Retic-ABS and NRBC (p < 0.001) as compared to the normoxia. The combined IHT and ACZ group maintained a significantly lower hematological values with respect to all the parameters (p < 0.01) as compared to the other groups exposed to extreme hypoxia, maintaining a reduced pathological changes in hemodynamics. The differential leukocyte expression in all the groups of animals are represented as scatter gram for each of the representative samples in Fig. 3(a–e).). Level of lymphocyte, monocyte and neutrophil expression was found to be highest in the EH group, while the combined effect of IHT and ACZ showed to maintain an average level. Multiple comparisons among groups for the changes in hematology were done through one way anova, depicted in the Table 2 and Fig. 4(A-K).

**Table 2:** Effect of IHT and ACZ on hematological parameters of extreme hypoxia exposed rats at 8% FiO2. Rats were pretreated with acetazolamide and subjected to intermittent hypoxia training at 12% FiO_2_ followed by validation at 8% FiO_2_. Values are mean ± SD (n = 5). WBC (White Blood Cell), LYM % (Lymphocyte), MON% (Monocyte), NEU% (Neutrophil), RBC (Red Blood Cell), HB (Hemoglobin), HCT% (Hematocrit %), MCV (Mean Corpuscular Volume), PLT (Platelet), Retic (Reticulocyte %), Retic-ABS (Absolute reticulocyte count), NRBC (Nucleated RBC). Ns, Non-significant difference.

Parameters	N	IHT + EH	IHT + ACZ + EH	ACZ + EH	EH	Level of Significance
WBC	10.20 ± 2.13	12.20 ± 2.66	11.12 ± 1.66	12.60 ± 2.15	17.15 ± 2.00	≤0.0001
LYM %	73.51 ± 7.78	81.13 ± 13.04	78.70 ± 7.62	76.48 ± 11.16	93.16 ± 5.36	≤0.05
MON %	3.34 ± 1.32	5.55 ± 1.38	6.12 ± 2.46	5.82 ± 1.97	6.78 ± 1.41	ns
NEU %	17.43 ± 7.98	39.85 ± 19.38	22.38 ± 8.85	36.19 ± 16.54	54.67 ± 12.07	≤0.05
RBC 10^6^/ul	6.83 ± 0.96	8.83 ± 0.31	7.27 ± 0.65	8.35 ± 0.34	11.56 ± 0.75	≤0.0001
HB g/dL	10.67 ± 0.90	15.21 ± 0.80	15.19 ± 0.67	16.83 ± 0.39	19.21 ± 1.43	≤0.0001
HCT %	34.07 ± 5.53	37.22 ± 2.34	40.25 ± 3.99	48.56 ± 1.24	63.92 ± 4.18	≤0.0001
MCV fL	55.82 ± 1.48	64.43 ± 1.38	55.43 ± 1.66	58.22 ± 1.43	88.13 ± 1.19	≤0.0001
PLT 10^3^/ul	554.79 ± 107.46	678.52 ± 84.44	816.16 ± 160.80	988.50 ± 129.38	1092.20 ± 71.09	≤0.0001
Retic %	8.41 ± 1.04	13.77 ± 2.71	10.25 ± 3.79	13.34 ± 6.07	19.21 ± 4.75	≤0.05
Retic-ABS 10^9/L	530.07 ± 54.89	938.91 ± 177.94	784.91 ± 284.34	943.19 ± 337.36	1473.63 ± 475.10	≤0.05
NRBC %	2.46 ± 9.16	47.83 ± 27.34	36.57 ± 17.66	55.61 ± 21.88	89.05 ± 33.23	≤0.001

*p < 0.05, #p < 0.001, $p < 0.0001.

### 3.4 Alteration in the blood gas parameters under extreme hypoxia exposure

**Fig. 5:**
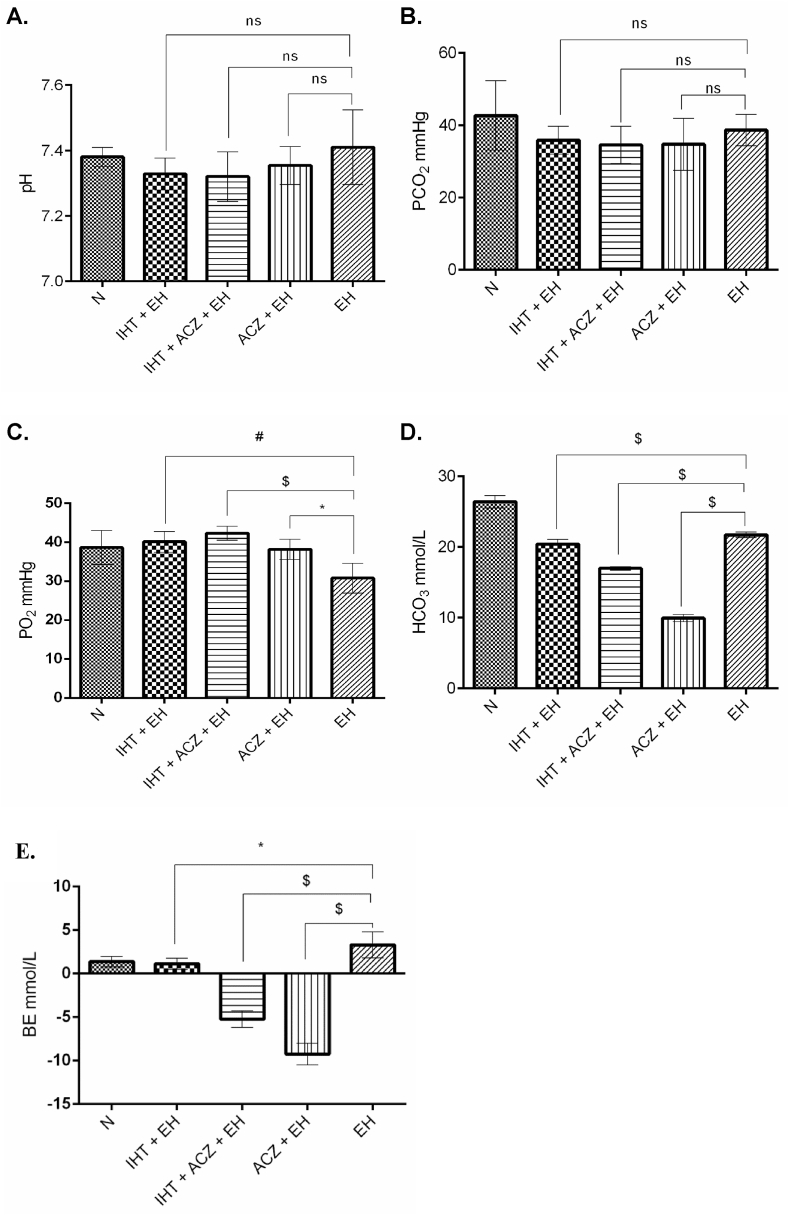
Graph representing comparison between the groups of Blood gas variables. Data was assessed by one-way analysis of variance (ANOVA) applying the Bonferroni correction for multiple comparisons among groups. N: Normoxia, IHT + EH: Intermittent hypoxia training + Extreme hypoxia, IHT + ACZ + EH: Intermittent hypoxia training + Acetazolamide + Extreme hypoxia, ACZ + EH: Acetazolamide + Extreme hypoxia, EH: Extreme hypoxia **A.** pH, **B.** PCO_2_ (mmHg), **C.** PO_2_ (mmHg), **D.** HCO^−^_**3**_ (%), **E.** BE (mmol/L) *p < 0.05 #p < 0.001 $p < 0.0001, values are mean ± SD.

There was a significant decrease in blood PO_2_, HCO_3_^−^ and BE in all the extreme hypoxia exposed groups as compared to normoxia. Comparisons among the extreme hypoxia exposed groups were observed with a significantly higher PO_2_ (p < 0.001) and lower HCO_3_^−^ (p < 0.0001) and BE (p < 0.05) in the combined IHT and ACZ as compared to the EH group without any intervention. Multiple comparisons among groups for the changes in blood gasometry were done through one way anova, depicted in the Table 3 and Fig. 5(A-E).

**Table 3:** Effect of IHT and ACZ on venous blood gas at 8% FiO_2_ in rats. Rats were pretreated with acetazolamide and subjected to intermittent hypoxia training at 12% FiO_2_ followed by validation at 8% FiO_2_. Values are mean ± SD (n = 5). Ns, Non-significant difference.

Parameters	N	IHT + EH	IHT + ACZ + EH	ACZ + EH	EH	Level of Significance
pH	7.38 ± 0.03	7.33 ± 0.05	7.32 ± 0.08	7.35 ± 0.06	7.41 ± 0.12	ns
PCO_2_ mmHg	42.69 ± 9.59	35.85 ± 3.86	34.50 ± 5.15	34.70 ± 7.21	38.62 ± 4.35	ns
PO_2_ mmHg	47.58 ± 8.90	40.10 ± 2.68	42.29 ± 1.75	38.14 ± 2.59	30.75 ± 3.85	≤0.001
HCO_3_^−^ mmol/L	26.38 ± 0.89	20.39 ± 0.66	16.96 ± 0.24	9.92 ± 0.53	21.69 ± 0.40	≤0.0001
BE mmol/L	1.38 ± 0.60	1.12 ± 0.66	−5.24 ± 0.95	−9.24 ± 1.24	−3.30 ± 1.50	≤0.0001

*p < 0.05, #p < 0.001, $p < 0.0001, PCO_2_ mm Hg (Partial Pressure of Carbon Dioxide), PO_2_ mm Hg (Partial Pressure of Oxygen), HCO_3_^−^ mmol/L (Bicarbonate), BE mmol/L (Base Excess).

## 4 Discussion

When hypoxia increases during ascent to the high altitude, ventilatory control and the related oxygenation is compromised often leading to altitude related maladies. The lower partial pressure of oxygen (hypoxia) and raised partial pressure of CO_2_ (hypercapnia) in the arterial blood on exposure to high altitude, triggers a series of compensatory adjustments by various organ systems by stimulating the peripheral chemoreceptors along the aorta and the carotid sinus. In response to peripheral chemoreceptors, the respiratory centre in the hypothalamus is triggered and the signals are relayed to the diaphragm, intercostal muscles and stretch receptors of the lungs to facilitate hyperventilation. Hyperventilation is the first and foremost response to high altitude exposure which is thus achieved by hypoxic ventilatory response of the peripheral chemoreceptors (San et al., 2013). Acetazolamide is the drug of choice for prophylaxis against AMS stimulating hyperventilation (Paralikar and Paralikar, 2010).

In the present study, we investigated the combined effect of pharmacological (acetazolamide) and non-pharmacological (Intermittent hypoxia training) approaches to mitigate the effects of hypoxia related maladies under simulated hypoxic conditions. To determine the combined effect of acetazolamide and intermittent hypoxia training, lowest effective dose and a previously studied IHT protocol were used (Lipman et al., 2019; Nimje et al., 2020). Altitude acclimatization is a series of physiological responses that compensate for the reduction in the partial pressure of oxygen with increasing altitude. Several studies show higher oxygen saturation level is associated with hypoxic acclimatization and thus oxygen saturation monitoring can serve as a relevant indicator of acclimatization to high altitude (Babcock and Newberry, 2014; Karinen et al., 2010; Roach et al., 1998). In this study, the combination of intermittent hypoxia training and ACZ was observed to maintain a higher and uniform SpO_2_ level, indicating that the oxygen desaturation due to hypoxia exposure was best prevented by the administration of ACZ along with IHT.

Intermittent hypoxia has been reported to increase the hypoxic ventilatory response which could be associated with an augmented sensory response of peripheral chemoreceptors to acute hypoxia, thereby maintaining higher SpO_2_ (Ainslie et al., 2007; Peng et al., 2001). On the other hand, acetazolamide administration stimulates the metabolic acidosis and increases the ventilatory response to hypoxia mainly through central chemoreceptors and thus improves pulmonary gas exchanges (Swenson, 1998). Previous studies have reported increased arterial oxygen saturation after pre-administration of acetazolamide, which assisted in ascent to Everest base camp and Mt Kilimanjaro (Bradwell et al., 1981; Greene et al., 1981). This possibly explains that intermittent hypoxia and ACZ, in combination, improves oxygen saturation under hypoxic condition by maintaining a higher SpO_2_ level.

Limited information is available on combined effect of IHT and acetazolamide. So far, only one study on arterial oxygen saturation of human subject, pre-treated with IH and acetazolamide, have been reported (Burtscher, 2008). The author observed prevention of oxygen desaturation along with prolonged and constant SaO_2_ periods in the human subject exposed to high altitude. However, that study cannot be compared with the present study as it was conducted on a single human subject and the measurement of a single physiological parameter (arterial oxygen saturation) was done during the ascent to high altitude.

Besides decreasing oxygen saturation, altitude exposure causes changes in heart rate and respiration rate (Chawla and Saxena, 2014). Exposing the rats to 8% FiO_2_ resulted into an increase in heart rate in all the groups. The increase in HR during the hypoxia exposure signifies the immediate compensatory response of the cardiopulmonary system to oxygen desaturation in order to maintain physiological homeostasis. The HR in the IHT + ACZ treated group was found to be maintained in a flattened curve as compared to the other groups. Similar observations were made by previous researchers in human subjects, which showed that heart rate at high altitude was maintained at a lower level in subjects administered with acetazolamide (Bradwell et al., 2018; Parati et al., 2013). However, other reports on intermittent hypoxia reveal contrasting effects on HR and heart rate variability (HRV). Some studies showed that IHT did not alter HR (Julian et al., 2004), and heart rate variability (Board et al., 2012), whereas other studies showed that IHT could increase HRV (Teppema et al., 2007). Thus, the effect of IHT on changes in HR is not constant as observed from our study as well as previous study reports. The reason behind contrasting results in HR and HRV is not well understood.

Respiratory rate was found to be maintained at a highest level in the animals exposed to extreme hypoxia followed by the ACZ administered group alone. It can be speculated that the moderate increase in RR may be beneficial in contrast to the sudden rise in RR as observed in the extreme hypoxia group.

Investigations on the hypoxia induced changes in hemodynamics and blood gasometry revealed that extreme hypoxia exposure of rats to 8% FiO_2_ caused an increase in all the hematological variables, viz., leukocytes, RBC, HCT, PLT and reticulocytes as a part of hematological adjustments for increased tissue oxygenation. Previous studies reported alterations of hematological variables due to high altitude hypoxia (Alvarez-Martins et al., 2016; Cowburn et al., 2017; Robergs and Roberts, 2000).

Optimum hematocrit level for tissue oxygenation under hypoxic conditions is of great importance for acclimatization at high altitude. There was moderate rise in RBC, Hb, HCT and PLT in the combined IHT and ACZ group in the present study. Previous studies using the acetazolamide as either preventive or curative treatment under hypoxic exposure revealed that acetazolamide is effective in decreasing blood viscosity, reduction in pulmonary vascular resistance as well as reduction in right ventricular hypertrophy in rats (Pichon et al., 2012). Hematological adaptation to high altitude is associated with an increase in hematocrit values that is attributed to a shift of water out of the vascular system leading to an increase in haemoglobin concentration that enables the body to compensate oxygen dependent energy deficit (Mason, 2000; McArdle et al., 2010). Hematocrit levels are increasing in all the hypoxia exposed group. Under normal pulmonary function the linear increase in hematocrit is associated with an increase in arterial oxygen content (CaO_2_) and thus oxygen delivery (DO_2_) (Messmer, 1994). However, it should be noted that delivery of oxygen is affected by hemoconcentration and polycythemia. Thus an optimal level of hematocrit is linked to better oxygen delivery (Reinhart, 2016). Reticulocyte count is considered to be a sensitive indicator of tissue hypoxia (Takagi et al., 2017). In the present study, rise in reticulocytes and nucleated RBCs was observed in all the treated groups of animal. The rise in reticulocytes and immature nucleated RBC could be due to hypoxia, leading to the activation of hypoxia-inducible transcription factors (HIF-1α), which, in turn, might initiate the production of erythropoietin (EPO) and, thereby, increase in the reticulocyte count (Ostergaard et al., 2014).

Neutrophil % (NEU %) was observed to increase in all the hypoxia exposed groups. NEU % was highest in the group exposed directly to extreme hypoxia, indicating inflammatory response. Similar finding was reported in the previous work of Schoene et al. (1988) showing neutrophilia in alveolar lavage fluid of HAPE patients. In the present study, the increase in NEU % might be due to stabilized HIF-1α expression that occurs during hypoxia. HIF-1α is said to increase the lifespan of neutrophils in peripheral blood by inhibiting its apoptosis via PI3 kinase dependent survival factor (Walmsley et al., 2005).

In our study, the exacerbated rise in reticulocyte and nucleated RBC counts in EH group was managed and controlled by the combined effect of IHT and ACZ treatment. Thus, evidences from the previous studies as well as present study suggest that pre-treatment with IHT and ACZ could prevent hematological pathologies caused due to extreme hypoxia.

Exposure of the rats to extreme hypoxia at 8% FiO_2_ also resulted into substantial changes in the blood gas parameters in all the groups of rats. In the combined IHT and ACZ group as compared to the EH group, there was reduction in pH and pCO_2_, significant increase in pO_2_, and decrease in HCO_3_^−^ and BE. This could be better explained from previous studies that acetazolamide administration generates metabolic acidosis by increasing the excretion of bicarbonate (HCO_3_^−^), which causes reduction in base excess (BE). The reduced base excess (BE), in turn, attenuates the effects of respiratory alkalosis by reducing the blood pH and enhancing ventilation (West, 2004) allowing peripheral chemoreceptors to fully respond to low PaO_2_ (Cain and Dunn, 1965). Thus, the IHT + ACZ in combination maintained higher blood pO_2_ level by compensating the blood gas parameters.

**Fig. 6:**
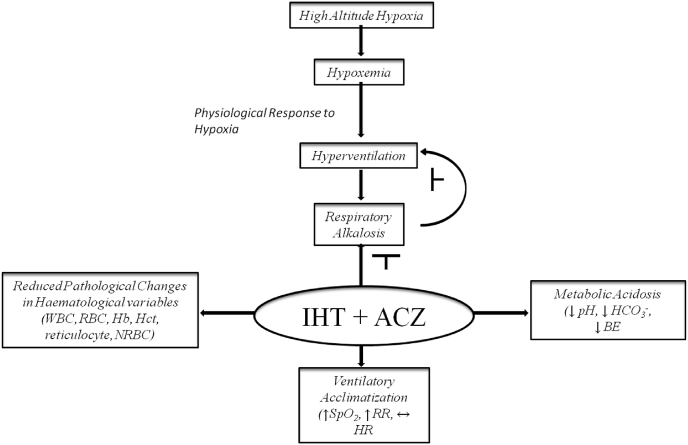
Schematic representation for the possible mechanism of combined effect of Intermittent hypoxia training (IHT) and Acetazolamide (ACZ), as a prophylactic strategy for high altitude acclimatization. ↑Increase, ↓ Decrease, ↔ Stabilized. ˫ Attenuation.

Taken together from previous findings as well as the present study, a schematic illustration representing hypothesis and possible mechanism of combined effect of Intermittent hypoxia training (IHT) and Acetazolamide (ACZ), as a prophylactic strategy for high altitude acclimatization is depicted in Fig. 6.

## 5 Conclusion

Individuals who are acclimatized to extreme altitude induced hypoxic stress shows extraordinary changes in the physiological, hematological and blood gasometry in response to the low inspired pO_2_. It has been observed from the present study that these responses include an increase in respiratory rate which maintains the alveolar pO_2_ as well as peripheral oxygen saturation (SpO_2_). The combined effect of intermittent hypoxia training and acetazolamide administration as a prophylactic agent showed the consequent metabolic acidosis evidencing reduced pH, bicarbonate and base excess, which in turn might have attenuated the inhibitory effects of hypoxia induced respiratory alkalosis owing to hyperventilation. All these events might have increased the oxygen affinity of hemoglobin elevating the SpO_2_ level under extreme hypoxic condition. Thus, the combined effect of IHT and ACZ may help in devising better therapeutic modality for altitude acclimatization in the subject ascending to high altitude.

However all these physiological events in response to extreme hypoxic conditions should be thought of in terms of survival rather than successful long term adaptation to the high altitude hypoxic environment, as both IHT and Acetazolamide effects has got its own limited withdrawal time period, which needs to be further investigated.

## Limitations

The multichannel pulse oximetry data recording system (Starr Life sciences, Mouse Ox, USA) assembled with the normobaric hypoxia chamber (Biostag Technologies Limited, India) that was used in the present study could measure physiological parameters of only 5 numbers of animals at a time. Therefore, the recording of physiological parameters of all the groups could be accomplished after conducting number of experiments.

## CRediT authorship contribution statement

**Megha A. Nimje:** Investigation, Data curation, Writing – original draft, Visualization, Formal analysis. **Himadri Patir:** Conceptualization, Methodology, Investigation, Validation, Writing – review & editing. **Rajeshkumar Tirpude:** Conceptualization, Investigation, Writing – review & editing. **Bhuvnesh Kumar:** Supervision, Resources, Writing – review & editing.

## Declaration of competing interest

The authors declare that they have no known competing financial interests or personal relationships that could have appeared to influence the work reported in this paper.

## Abbreviations

IHT: Intermittent hypoxia training
ACZ: acetazolamide
FiO_2_: Fraction of inspired O_2_
EH: extreme hypoxia
SpO_2_: Peripheral oxygen saturation
HA: high altitude
AMS: acute mountain sickness
N: Normoxia
DMSO: Dimethyl sulfoxide
LD50: Lethal Dose 50%
HR: Heart rate
RR: Respiratory rate
CBC: Complete Blood count
PCO_2_: Partial pressure of Carbon Dioxide
PO_2_: Partial pressure of oxygen
BE: Base Excess
HCO_3_^−^: Bicarbonate
ANOVA: one-way analysis of variance
WBC: White Blood cells
Lym: Lymphocytes
Mon: Monocytes
Neu: Neutrophils
RBC: Red Blood cells
Hb: Hemoglobin
HCT: Hematocrit
MCV: Mean Corpuscular Volume
PLT: Platelet
Retic: Reticulocytes
Retic-ABS: Reticulocyte absolute
NRBC: Nucleated Red blood cells
HRV: Heart rate variability
